# Terpene polyacrylate TPA5 shows favorable molecular hydrodynamic properties as a potential bioinspired archaeological wood consolidant

**DOI:** 10.1038/s41598-021-86543-1

**Published:** 2021-04-01

**Authors:** Michelle Cutajar, Fabrizio Andriulo, Megan R. Thomsett, Jonathan C. Moore, Benoit Couturaud, Steven M. Howdle, Robert A. Stockman, Stephen E. Harding

**Affiliations:** 1 National Centre for Macromolecular Hydrodynamics (NCMH), School of Biosciences, University of Nottingham, Sutton Bonington, LE12 5RD UK; 2 School of Chemistry, University of Nottingham, University Park Nottingham, NG7 2RD UK; 3 Museum of Cultural History, University of Oslo, St. Olavs plass, Postboks 6762, 0130 Oslo, Norway; 4 Univ Paris Est Creteil, CNRS, Institut de Chimie et des Matériaux Paris-Est (ICMPE), UMR 7182, 2 rue Henri Dunant, 94320 Thiais, France

**Keywords:** Biotechnology, Chemistry

## Abstract

There is currently a pressing need for the development of novel bioinspired consolidants for waterlogged, archaeological wood. Bioinspired materials possess many advantages, such as biocompatibility and sustainability, which makes them ideal to use in this capacity. Based on this, a polyhydroxylated monomer was synthesised from α-pinene, a sustainable terpene feedstock derived from pine trees, and used to prepare a low molar mass polymer TPA5 through free radical polymerisation. This polymer was extensively characterised by NMR spectroscopy (chemical composition) and molecular hydrodynamics, primarily using analytical ultracentrifugation reinforced by gel filtration chromatography and viscometry, in order to investigate whether it would be suitable for wood consolidation purposes. Sedimentation equilibrium indicated a weight average molar mass *M*_w_ of (4.3 ± 0.2) kDa, with minimal concentration dependence. Further analysis with MULTISIG revealed a broad distribution of molar masses and this heterogeneity was further confirmed by sedimentation velocity. Conformation analyses with the Perrin *P* and viscosity increment ν universal hydrodynamic parameters indicated that the polymer had an elongated shape, with both factors giving consistent results and a consensus axial ratio of ~ 4.5. These collective properties—hydrogen bonding potential enhanced by an elongated shape, together with a small injectable molar mass—suggest this polymer is worthy of further consideration as a potential consolidant.

## Introduction

Throughout history, people have been constructing artefacts and tools out of wood. Such archaeological discoveries provide us with a fascinating insight into the everyday life of that particular civilisation, as well as their ceremonial rituals and mastery of woodworking^1,2^. Unfortunately, wooden archaeological findings are relatively rare due to their biodegradability^3–5^ and are therefore considered to be very precious, requiring carefully tailored treatment strategies in order to conserve them for future generations. The degradation state of the archaeological wood depends on multiple factors such as its age, species and most importantly, its environment^4,6^. Common locations where archaeological wood discoveries are usually made include wet environments like bogs or the seabed^4,7^, as wood that is not in contact with oxygen has a slow degradation rate due to low microbial survival, as has been well reported^2,8^. An example is the Oseberg Viking Ship collection which was able to last thousands of years as it was buried in blue clay that provided a highly anaerobic environment^9^. Most of the artefacts found on the Oseberg ship were treated with alum (aluminium potassium sulfate dodecahydrate, KAl(SO_4_)_2_·12H_2_O) in the early twentieth century^10^, which is now known to have directly contributed to the artefacts’ current state of degradation^1,11^.

There is a pressing need for the development of new consolidant materials which may be used to treat archaeological waterlogged wood, especially unique cases like the Oseberg artefacts. Such consolidants should ideally be biocompatible and interact with the structure of the archaeological artefact, providing support without causing morphological distortion^12^. Potential consolidants must also penetrate the wood to an appropriate depth whilst leaving room in the pores for future re-treatment. Polymers which are to be used as consolidants are therefore required to have a low molar mass in order to be able to adequately penetrate the wood cells. As a point of reference, polyethylene glycol (PEG) usually has a molar mass ranging from 0.2 to 4.0 kDa when used for conservation^13^.

PEG has long been popular for treating archaeological wood and has notably been used for the conservation of famous shipwrecks like the Mary Rose^14^ and the Vasa^15^. Despite its popularity, there are several valid reasons why it is necessary to find an alternative. Not only is it petroleum-derived, but there is also the possibility that it may gradually degrade in wood, potentially leading to the release of formic acid^12,16^. Additionally, PEG is vulnerable to environmental factors such as high temperatures^15^, as well microbial degeneration^17^.

Walsh et al.^18^ note that biological based polymers offer several advantages and that they represent a greener and more sustainable alternative to some of the consolidants currently in use, such as PEG. Such materials have already been implemented successfully in different areas of scientific research, with Cabane et al.^19^ providing us with various examples including surface wettability; photonics; self-healing and composite mechanical reinforcing^20–24^. With regards to conservation, there have been numerous studies over the years surrounding the use of green materials as consolidants^25–31^. A consolidant should ideally have high hydrogen bonding and other interaction potential, through appropriate residues and enhanced by an elongated shape, together with a small injectable molar mass which after curing inside the wood makes a strong, slightly flexible polymer network which interacts with the remaining wood materials. Additionally, from an environmental perspective, it should ideally come from natural materials, i.e. be “bioinspired”: inspired by or based on biological structures or processes.

Terpenes are an example of such compounds which have recently generated considerable interest due to their potential as feedstocks for renewable polymers^32^. They are derived from a number of natural resources, such as trees and other plants^33^, and are especially interesting since they can be obtained from biomass like wood waste without competing with food production^34^. Apart from their sustainable nature, other advantages include the ability to lend unique structural features like functionalities and stereocentres to their resultant polymers^34–41^. Stereocentres provide three-dimensionality to the molecules which can improve their potential for interactions, possibly leading to an increase in strength. Such functionalisation makes these polymers highly tuneable and gives them useful characteristics, including the ability to form hydrogen bonding, networking with the wood structure and possible anti-microbial properties. The production of such materials - which must be small enough to penetrate the wood prior to interacting/ curing or networking with the wood structure - is a compelling route to investigate as these compounds may potentially be used as consolidants for archaeological objects like the Oseberg artefacts. It was therefore decided to focus on terpene-based polymers for this study, due to the numerous advantages that they offer. Up until the present, we have been unable to find any literature detailing research around the use of terpene-based compounds as consolidants in heritage conservation.

In this study, we describe the synthesis and polymerisation of a functionalised monomer derived from α-pinene. This is a particularly interesting monoterpene as it is highly abundant, being a major component of turpentine which is itself derived from pine resin^42^. As a result, it has already been the focus of numerous studies as detailed in a recent review by Thomsett et al.^34^ It was decided to furnish the monomer with two hydroxyl groups which would increase the potential for hydrogen bonding with the wood structure and consequently, its consolidative ability^43^. Extensive characterisation studies were subsequently run on the polymer, primarily using analytical ultracentrifugation. This is considered to be one of the most functional and adaptable techniques used in the study of macromolecules^9,44^. It is used for the characterisation of particles’ sizes and shapes, as well as the quantitative analysis of their interactions in solutions^9,44,45^. This instrument is particularly useful since it functions without the need of separation matrices and calibration standards^9^. Such a hydrodynamic study will allow us to evaluate the polymer’s potential as a consolidant for waterlogged, archaeological wood: finding favorable molecular hydrodynamic properties is very much Phase 1 in the process of finding a successful consolidant.

## Results and discussion

### Monomer synthesis from α-pinene

**Figure 1:**
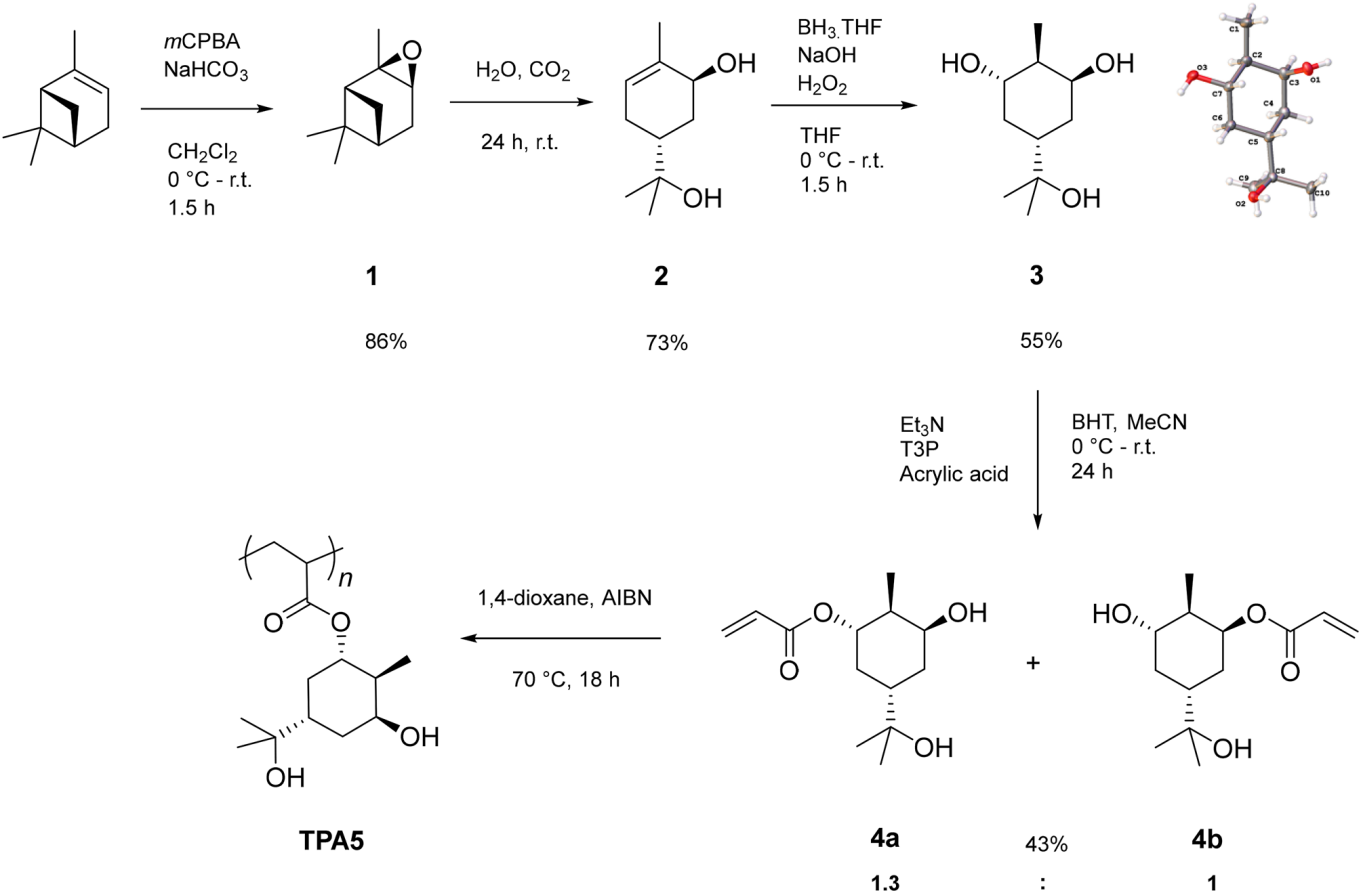
The synthesis route for the final monomer. An epoxidation of the starting terpene was carried out to form α-pinene oxide **1**, which was then hydrolysed to give *trans*-sobrerol **2**. This in turn was used to carry out a hydroboration/oxidation reaction to give the triol **3**, which was afterwards functionalised with an acrylate group to form the monomer as a mixture of two diastereomers **4a** and **4b**. These were then polymerised to form **TPA5**.

We have recently reported the synthesis of the terpene-derived triol **3** (Fig. 1) and its co-polymerisation with succinic acid via step-growth polymerisation^46^. The resulting polyesters were shown to be stereoregular; a feature that was exploited through the formation of a polymer stereocomplex^46^. As hydrogen bonding potential is anticipated to be a valuable property in wood consolidants, we were keen to investigate the polyhydroxylated derivative **3** for this purpose. Nevertheless, it was anticipated that the aforementioned polyesters would be biodegradable, a feature which is of course undesirable in this context. Accordingly, we instead investigated the formation of an acrylate derivative.

The synthesis of the acrylated monomer **4** was achieved in four steps from the cheap and readily available residue material α-pinene (Fig. 1 and also Supplementary Information for full synthetic details of **1**, **2** and **3**). The first step is epoxidation to the corresponding oxirane α-pinene oxide (**1**). The epoxidation of α-pinene has been the focus of extensive research (as detailed by Corma Canos et al.^46^) and it is currently synthesised on an industrial scale, as well as being widely available commercially. The next step involved forming *trans*-sobrerol (**2**), which is a common hydrolysis product of **1** and is a known molecule from our in-house library^35,47^.The triol **3** was synthesised from **2** using a Brown hydroboration/oxidation sequence^48^ and subsequently isolated as a single diastereomer, the stereochemistry of which was confirmed by X-ray crystallography. It should be noted that a higher yield for **3** can be achieved via column chromatography, at the expense of the diastereomeric ratio^46^. Polyhydroxylated compounds such as these have a lot of untapped potential, not only for wood consolidation purposes but also for other applications such as drug delivery^49^.

The final step in the monomer synthesis involved functionalising **3** with an acrylate moiety. Previous work^35^ has demonstrated the (meth)acrylation of a number of alcohols derived from several terpenes like (+)-α -pinene, (−)-β-pinene and (*R*)-(+)-limonene. The addition of a (meth)acrylate functional group is a well-known process widely employed to enable a variety of monomers to undergo free radical polymerisation (FRP). In one such example^35^, hydroxyl-functionalised terpenes were initially esterified with (meth)acryloyl chloride using triethylamine as a base. This route was further optimised by substituting the (meth)acryloyl chloride with (meth)acrylic acid. Although these acids are not currently renewable, it is likely they will both become commercially available from a sustainable source in the future^35^. Additionally, the use of (meth)acrylic acid generates ‘green’ and less toxic waste as opposed to chlorinated waste, making the reaction more sustainable and scalable.

Propylphosphonic anhydride (T3P) was used to promote the ester coupling between the acrylic acid and **3**. This reagent is particularly attractive since its by-product is a relatively harmless and water-soluble triphosphate, again a preferable alternative to chloride waste generated when using acryloyl chloride. The reaction resulted in a mixture of diastereomers **4a** and **4b**. The final product was successfully isolated in two separate fractions: one enriched in the major diastereomer **4a** and another enriched in the minor diastereomer **4b**. The fraction enriched in the major diastereomer **4a** was isolated in a yield of 19% with a ratio of **4a**:**4b** = 22.8:1. The fraction enriched in the minor diastereomer **4b** had a yield of 23% with a ratio of **4a**:**4b** = 1:4.6. The total yield of diastereomers **4a** and **4b** was therefore 43%, in a ratio of **4a**:**4b** = 1.3:1. The diastereomers **4a** and **4b** were used together as a mixture for the subsequent polymerisation attempts. Diastereomer **4a** shows a large 11 Hz coupling on the proton adjacent to the acrylated oxygen (which is upshifted from 3.5 to 4.8 ppm by the electron-withdrawing carbonyl), consistent with a coupling between two axial protons.

### Polymerisation of triol acrylate, TPA5

The triol acrylate monomer was then polymerised via conventional free radical polymerisation (FRP) to produce polymer **TPA5**, employing AIBN as an initiator and 1,4-dioxane as the solvent at 70 °C. The reaction was monitored using ^1^H NMR and gel permeation chromatography (GPC) in THF (Supplementary Fig. S1). The investigated polymerisation reached 46% monomer conversion after 24 h under these reaction conditions as determined by ^1^H NMR. **TPA5** was then isolated by precipitation in acetonitrile as a white solid, with subsequent analyses confirming that the desired polymer was obtained (Supplementary Fig. S2). GPC analysis confirmed a number average molar mass (*M*_n_) of 2.6 kDa, a weight average molar mass of 3.8 kDa (relative to polymethylmethacrylate standards) and a broad polydispersity (*Đ*) of 1.5. These results were promising for our application as the polymer obtained appeared to have a low molar mass which was highly suitable for wood penetration. Nevertheless, higher conversion and narrower polydispersities can potentially be envisaged by using controlled polymerisation techniques such as reversible addition—fragmentation chain-transfer polymerisation (RAFT) or atom transfer radical polymerisation (ATRP).

### Solubility testing

The severe decay of the Oseberg artefacts is such that most of them are only held together by the alum present in their wooden structure. The objects with the highest degree of degradation cannot be treated with polymers in aqueous solvents as has been done previously with the Mary Rose^50^ and the Vasa^15^. This is because the alum remaining in the artefacts may dissolve in aqueous solvents and consequently exit the wooden structure, leading to total disintegration. As a result, it is deemed preferable to treat the most deteriorated artefacts with polymers in organic solvents.

Before continuing with the characterisation studies, it was therefore essential to determine which organic solvents the polymer was soluble in. Solvents which were tested included isopropanol, a 1:1 mixture of toluene in ethanol, ethyl acetate and acetone. The polymer was found to be soluble in isopropanol and the ethanol/toluene mixture, but insoluble in both ethyl acetate and acetone. It was therefore decided to carry out all further characterisation studies in isopropanol as this solvent has already been used in conservation studies and its high volatility ensures that it does not interact with the wood^51,52^. Furthermore, it has a low surface tension which allows for total permeability of the wood cells^52^.

### Calculation of the partial specific volume $\bar v$

**Figure 2:**
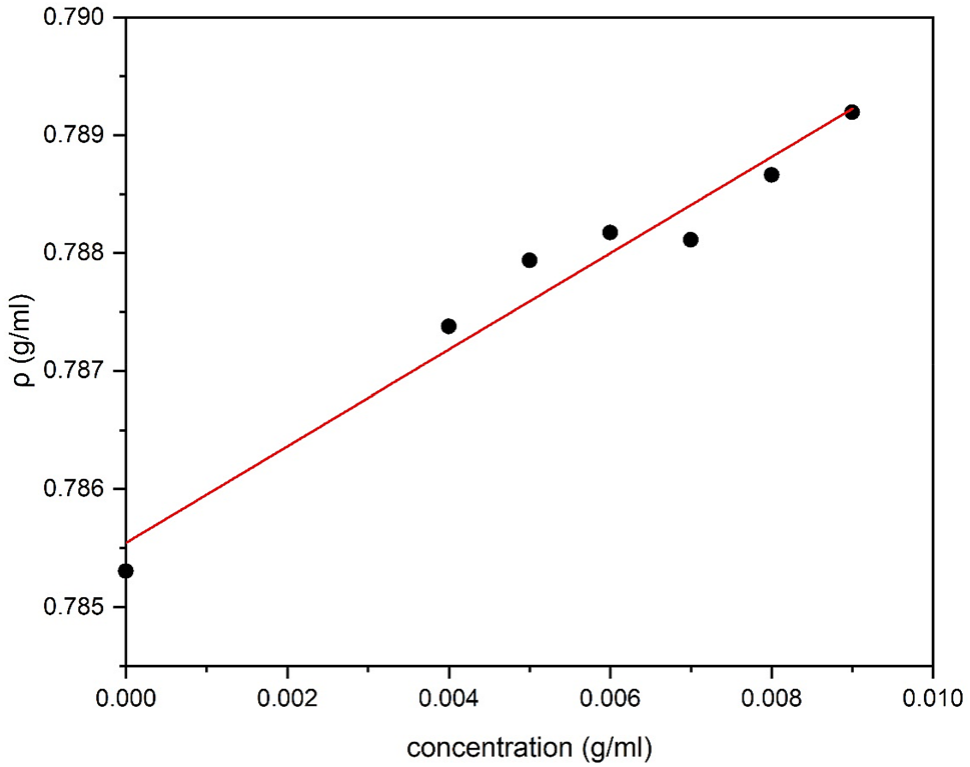
Solution density of **TPA5** in isopropanol plotted against concentration.

We essentially followed the method of Kratky et al*.*^53^ The density measurements were carried out using a concentration series of **TPA5** in isopropanol. The $\bar v$ was then obtained by plotting all the density measurements against concentration (Fig. 2) and consequently making use of Eq. 1. This was calculated to be (0.753 ± 0.040) cm^3^/g. This value appeared to be consistent with other $\bar v$ values previously reported for synthetic polymers such as polymethacrylate^54^, and was subsequently used for the sedimentation velocity and equilibrium analyses.

1 $$\bar v=\frac{1}{{\rho }_{o}}(1-\frac{\partial \rho }{\partial c})$$

### Molar mass determination and distribution

**Figure 3:**
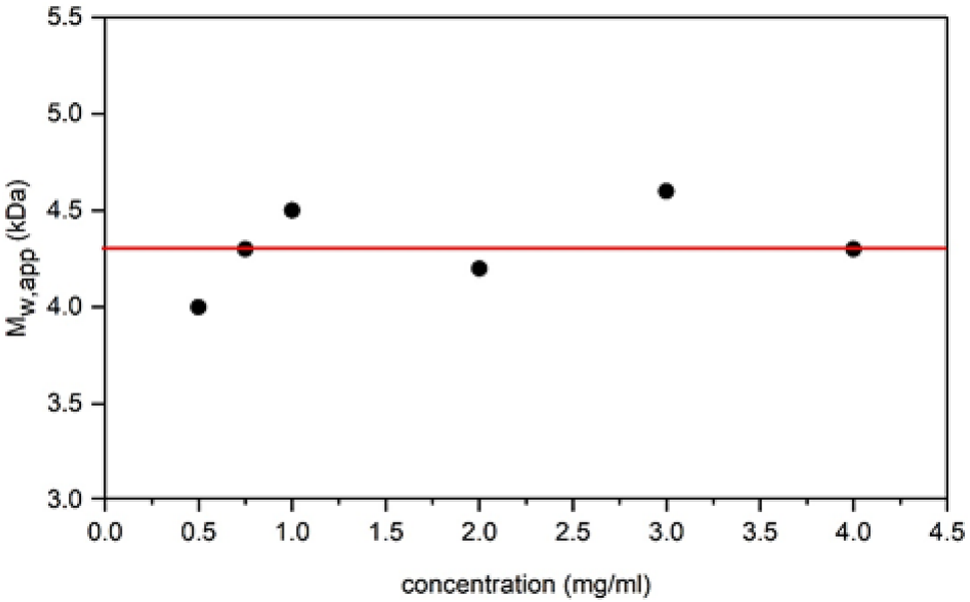
A plot of the apparent weight average molar mass, *M*_w,app_ against concentration, from sedimentation equilibrium. The *M*_w,app_ values were not significantly concentration-dependent, meaning that they were not affected by non-ideality. The ideal value was therefore taken as the average (red line), *M*_w_ = (4.3 ± 0.2) kDa. The data was analysed with SEDFIT-MSTAR using the M* method to obtain the shown *M*_w,app_ values. Rotor speed = 45,000 rpm.

Various loading concentrations were used to run the sedimentation equilibrium experiment in order to determine whether the measured or apparent molar mass *M*_w,app_ was dependent on concentration. The data was analysed with SEDFIT-MSTAR^55,56^, resulting in a plot of *M*_w,app_ vs concentration for each sample. The *M*_w,app_ was obtained by extrapolating the M* function to the cell base, as well as by using the hinge point method (Table 1). The ‘hinge point’ is defined as the radial position where the local concentration is equal to the initial loading concentration^55,56^. This analysis also provided information on the apparent z-average molar mass *M*_z,app_, which along with the *M*_w,app_ could then be used to calculate *Đ*. Table 1 shows a comparison of the *M*_w,app_, *M*_z,app_ and *Đ* values obtained from the sedimentation equilibrium study for all the tested concentrations. These results demonstrated that there was no significant change in *M*_w,app_ with concentration, indicating that it was not concentration dependent and not affected by non-ideality. It may therefore be assumed that the *M*_w,app_ determined by this experiment is in fact the ideal molar mass of the polymer at each given concentration (*M*_w,app_ ~ *M*_w_). The overall average *M*_w_ was calculated by plotting the values obtained in the sedimentation equilibrium experiment against concentration (Fig. 3). This was determined to be (4.3 ± 0.2) kDa.

**Table 1:** The *M*_w,app_ and *Đ* values obtained from the sedimentation equilibrium experiment for all concentrations.

Concentration (mg/mL)	*M*_w,app_ (from M*) (kDa)	*M*_w,app_ (hinge point) (kDa)	*M*_z,app_ (kDa)	*Đ* (*M*_z,app_/*M*_w,app_)
0.5	4.0	3.5	5.4	1.4
0.75	4.3	3.8	5.7	1.3
1	4.5	4.2	5.7	1.3
2	4.2	3.8	5.6	1.3
3	4.6	4.4	5.6	1.2
4	4.3	4.0	5.4	1.3

The *M*_w,app_ values obtained by the hinge point analysis were all slightly lower than the *M*_w,app_ (M*) values.

The *M*_w_ values obtained by sedimentation equilibrium and the GPC analysis that was previously carried out were compared, with the *M*_w_ obtained by sedimentation equilibrium proving to be slightly larger. It was expected that the values obtained from AUC would have a higher degree of accuracy as a result of it being an absolute method, meaning that it is matrix-free and does not require calibration standards.

**Figure 4:**
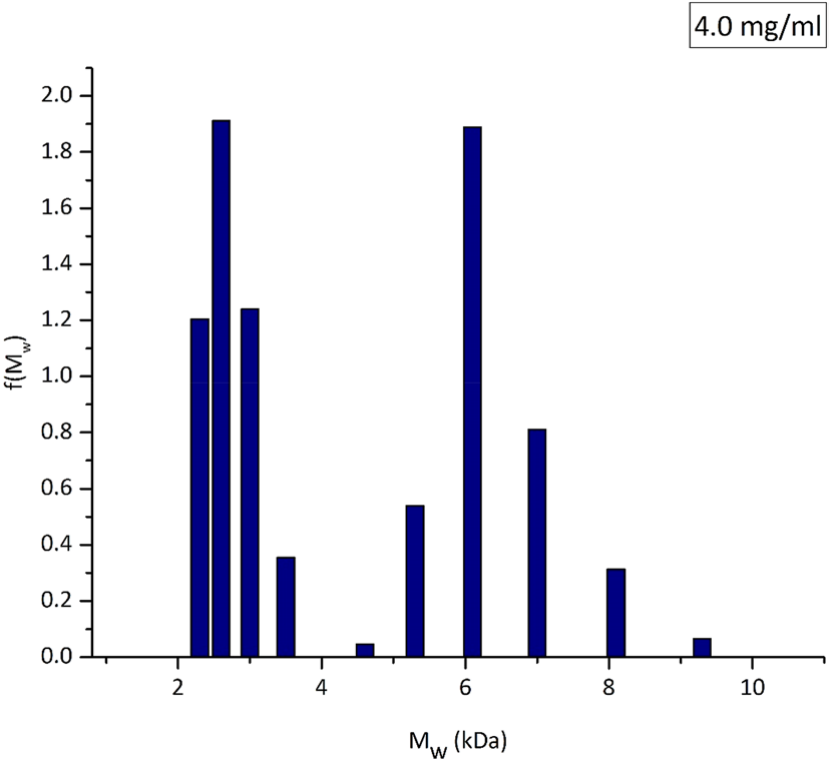
MULTISIG analysis of sedimentation equilibrium data to give the molar mass distribution f(*M*) vs *M*_w_ of **TPA5** at a concentration of 4.0 mg/mL. Rotational speed = 45,000 rpm.

The sedimentation equilibrium data for the 4.0 mg/mL concentration was additionally analysed with the MULTISIG algorithm. This programme provides a distribution of the molar masses by making use of a 17-component system with 20 iterations, assuming thermodynamic ideality^57^. It was used to give a broader resolution of the M* data, while also providing information about the *M*_w_ distribution of the polymer system. Figure 4 revealed a *M*_w_ distribution ranging from 2.3 to 9.3 kDa, with components peaking at 2.6 and 6.1 kDa. This was consistent with the SEDFIT-MSTAR analysis, confirming that the polymer has a low *M*_w_ and thus adding credibility to the results previously obtained by GPC. This further reinforced the status of this polymer as a very promising lead for the purpose of consolidation, since a low weight average *M*_w_ of ~ 4.3 kDa would increase the probability of it successfully penetrating archaeological wood.

### Determining the heterogeneity of the polymer system

**Figure 5:**
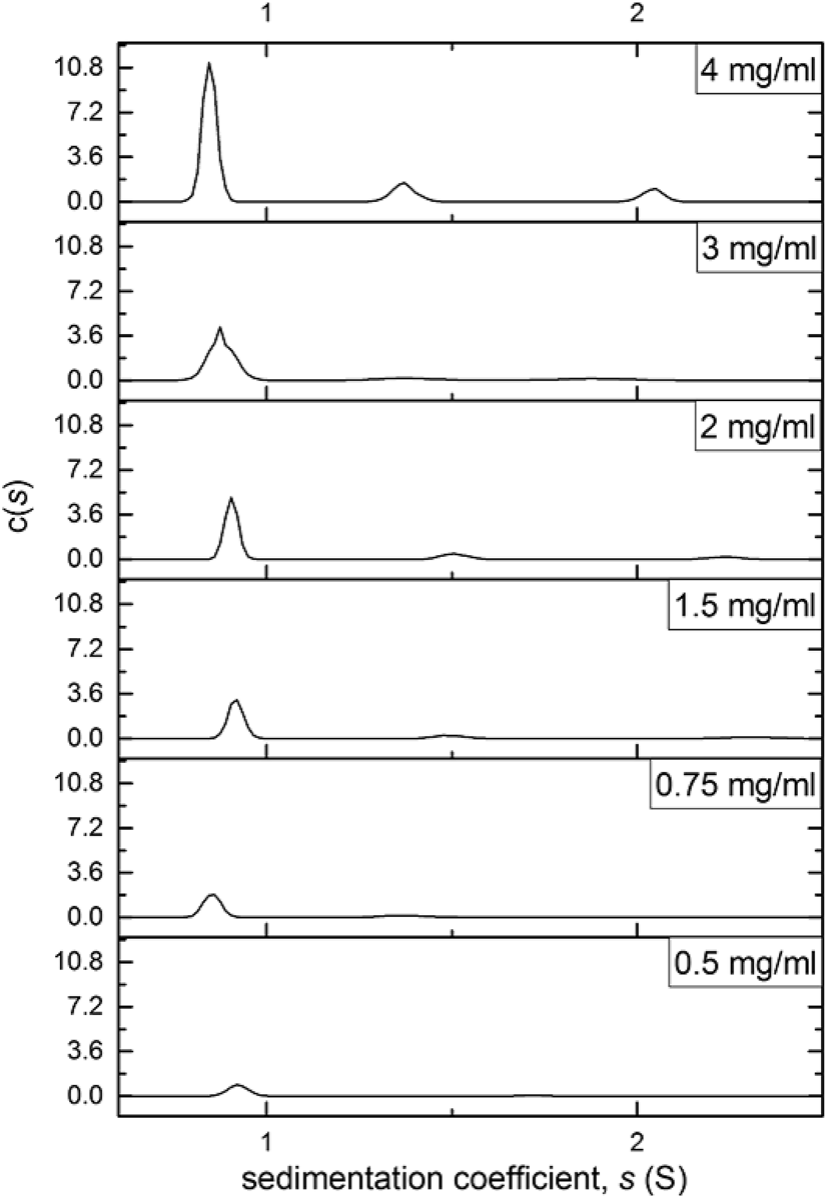
Sedimentation velocity sedimentation coefficient distributions c(*s*) vs *s* for **TPA5** in isopropanol. Distributions obtained by the SEDFIT approach of Dam and Schuck^61^. Rotor speed = 49,000 rpm.

Figure 5 shows the sedimentation coefficient range *c(s)*^58^ vs s of the polymer in isopropanol, run on SEDFIT. This algorithm normalises the sedimentation coefficient values to standard conditions (density and viscosity of water at 20.0 °C)^59^. The analysis confirmed that the polymer system is comprised of different components, as previously demonstrated by the MULTISIG analysis. It revealed the presence of a high concentration of a very low molar mass species, with a smaller population of two larger sedimentation coefficient, *s* value species. This trend was observed in all the concentrations that were used in this study.

### Calculation of the intrinsic viscosity $\left[{\varvec{\eta}}\right]$

It was initially planned to carry out the viscosity measurements of **TPA5** with a conventional Ostwald U-tube capillary viscometer^60^. This however did not prove ideal due to the high volatility of isopropanol. It was therefore decided to use a rolling ball viscometer at 8.0 °C in order to counteract this issue. After measuring the viscosity of the polymer solution at 6.0 mg/mL, the $\left[\eta \right]$ was calculated to be (5.27 ± 0.11) mL/g with the Solomon-Ciuta equation (Eq. 2).

2 $$\left[\eta \right]= \frac{1}{c}{\left(2\left({\eta }_{sp}\right)-2ln({\eta }_{r})\right)}^\frac{1}{2}$$

### Conformation analyses

With the use of $\left[\eta \right]$, along with the values for the *M*_w_, $\bar v$ and sedimentation coefficient obtained from previous experiments, the conformation of the polymer could then be investigated. This is particularly important as its shape may determine whether it penetrates wood and the subsequent effect it would have.

The programme ELLIPS1 was used to estimate the conformation of the polymer using the Perrin function (*P*) and the viscosity increment (ν) shape factors. ELLIPS1 is based on a simple ellipsoid of revolution model, where two of the three ellipsoid axes are of equal value^62^. Both *P* and ν are universal shape parameters, meaning that they are described by a function of shape not size^63^, and are obtained respectively from the sedimentation coefficient and $\left[\eta \right]$^64^.

*P* is related to the frictional ratio $(f/{f}_{o})$, which can be derived from the sedimentation coefficient *s*_*20,w*_ (Eq. 3)^65^:

3a $$\frac{f}{{f}_{o}}=\frac{M\left(1-\bar vp\right)}{{N}_{A}6\pi {\eta }_{o}}{\left(\frac{4\pi {N}_{A}}{3\bar vM}\right)}^\frac{1}{3}\frac{1}{{s}_{20,w}^{o}}$$

3b $$P= {(f/{f}_{o}) (\bar v/{v}_{s})}^\frac{1}{3}$$

ν is described by Eq. 4:^66–68^

4 $$\upnu = \left[\eta \right]M/\left({N}_{A}\mathrm{V}\right)$$

**Figure 6:**
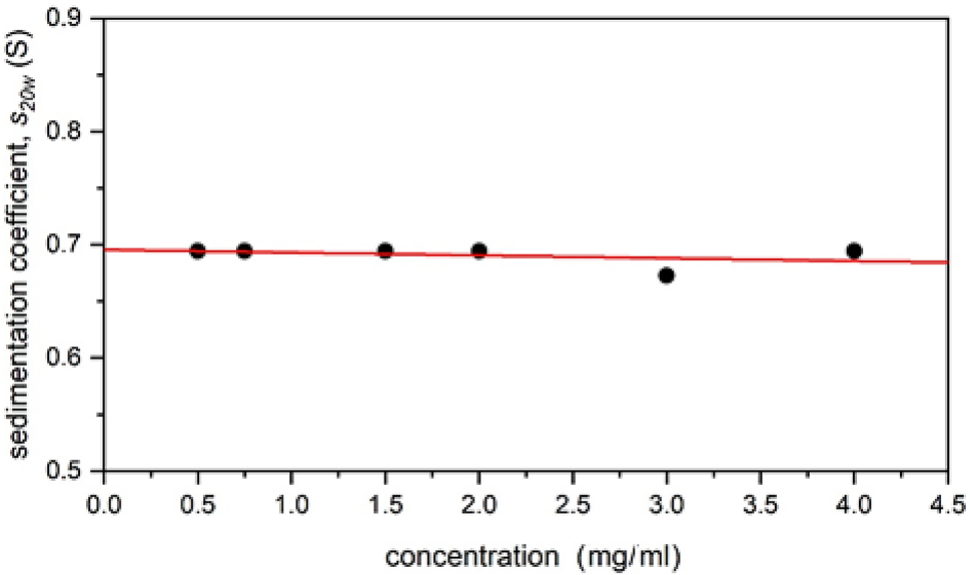
A plot of sedimentation coefficient *s*_20,w_ (corrected to standard conditions—the density and viscosity of water at 20.0 °C) against sedimenting concentration. The ideal or “infinite dilution” value, *s*^o^_20,w_ = (0.696 ± 0.007) S.

**Figure 7:**
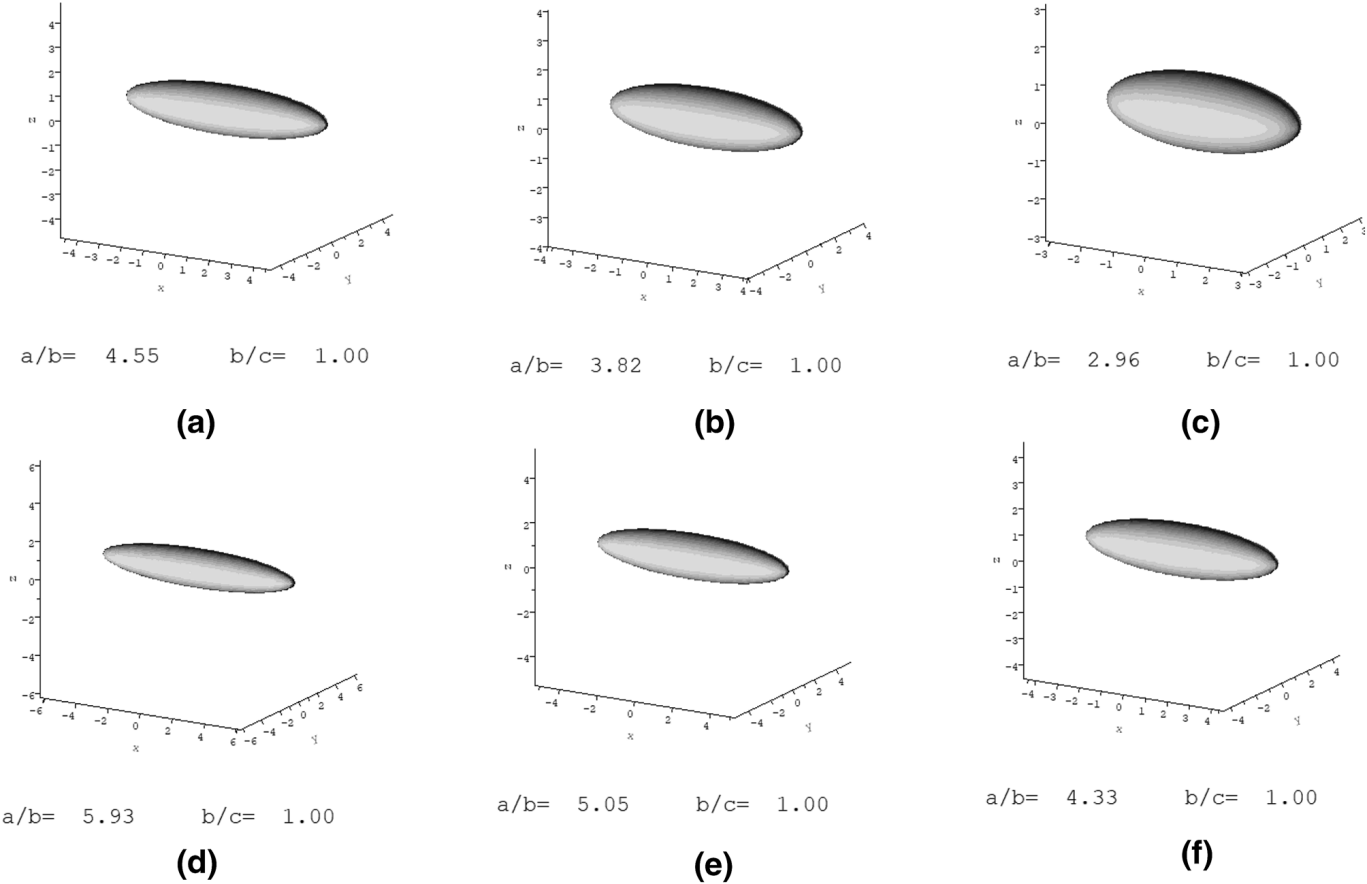
Ellipsoidal representations from the conformation analyses of **TPA5** in isopropanol, using ELLIPS1. (**a**) *P* for a $\left({\mathrm{v}}_{\mathrm{s}}/\bar v\right)$ = 1.0; (**b**) *P* for a $\left({\mathrm{v}}_{\mathrm{s}}/\bar v\right)$ = 1.2; (**c**) *P* for a $\left({\mathrm{v}}_{\mathrm{s}}/\bar v\right)$ = 1.4; (**d**) ν for a $\left({\mathrm{v}}_{\mathrm{s}}/\bar v\right)$ = 1.0; (**e**) ν for a $\left({\mathrm{v}}_{\mathrm{s}}/\bar v\right)$ = 1.2; (**f**) ν for a $\left({\mathrm{v}}_{\mathrm{s}}/\bar v\right)$ = 1.4. The data is consistent with an elongated shape in all the analyses.

Once calculated, ELLIPS1 was then used to report these shape functions in terms of their axial ratios $(a/b)$ for ellipsoids of revolution^63^. Table 2 shows the values obtained for these parameters. A sedimentation coefficient (*s*_20_*,*_w_) - normalised to the standard solvent conditions of the viscosity and density of water at 20.0^o^C  - was used in these calculations (Fig. 6). Along with the axial ratios, ELLIPS1 also provides a visual representation of the approximate shape of the macromolecule. The shape factors were calculated using different degrees of ‘solvation’ or solvent association or “dynamic binding” $\left({v}_{s}/\bar v\right)$, in order to determine whether the final value estimate for the type and axial ratio was significantly affected. Both *P* and ν were seen to alter slightly to higher or lower values according to the $\left({v}_{s}/\bar v\right)$, however they both gave consistent results, indicating that the polymer had an elongated shape, as shown by Fig. 7.

**Table 2:** The calculated values for the two different shape parameters *P* and ν at different degrees of solvent association (dynamic binding), along with the axial ratios determined by ELLIPS1.

Degree of solvent association $\left({v}_{s}/\bar v\right)$	Shape factor	Calculated value	Axial ratio $(a/b)$ (prolate)	Axial ratio $(a/b)$ (prolate) + error	Axial ratio $(a/b)$ (prolate)—error
1	*P*	(1.22 ± 0.20)	4.6	4.8	4.4
	$\upnu$	(7.00 ± 0.23)	5.9	6.1	5.7
1.2	*P*	(1.17 ± 0.20	3.8	4.0	3.6
	ν	(5.86 ± 0.23)	5.1	5.3	4.9
1.4	*P*	(1.11 ± 0.20)	3.0	3.2	2.8
	$\upnu$	(5.02 ± 0.23)	4.3	4.5	4.1

The axial ratios obtained by adding or subtracting the error from the calculated value (± 0.20 for *P* and ± 0.23 for ν) are also shown.

A consensus value of ~ 4.5 was obtained from the mean of the all the determinations. An additional shape factor, the Scheraga-Mandelkern ($\beta$) parameter, was used as a consistency check. This is a hydration-independent function, but it is very insensitive to shape (Eq. 5):^69^

5 $$\beta ={{N}_{A}s\left[\eta \right]}^\frac{1}{3}{\eta }_{o}/\left[{{M}_{r}}^\frac{2}{3}\left(1-{\bar v}\rho _{o}\right){100}^\frac{1}{3}\right]$$

**Figure 8:**
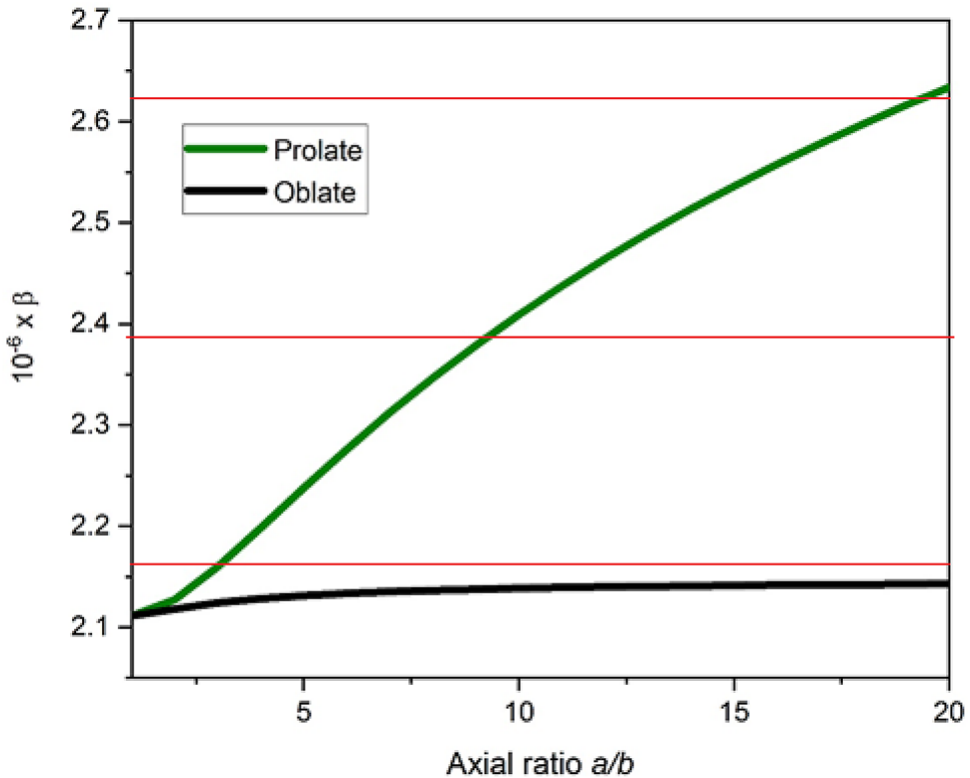
A plot of Scheraga-Mandelkern β function versus axial ratio with the green and black lines representing prolate and oblate shapes respectively. Red lines correspond to the experimental value of β = (2.39 ± 0.23) × 10^6^ which excludes an oblate (disc) shape.

A value of β = (2.39 ± 0.23) × 10^6^ was calculated. This corresponded to a large axial ratio range, but it was nonetheless consistent with the value for the *a*/*b* found for the *P* and ν functions. Additionally, this value is only compatible with an elongated, prolate ellipsoid molecule, as opposed to a flat disc or oblate model like lignin (Fig. 8). This therefore adds further confidence to the shape analyses that were previously carried out.

## Conclusion

In this study, the successful synthesis of an acrylated triol monomer **4** has been described. A new protocol for the radical polymerisation of such acrylated terpene monomers was designed and implemented, successfully yielding polymer **TPA5**. This methodology may potentially also be applied to other types of (meth)acrylated monomers derived from different terpenes. The polymer proved to be soluble in isopropanol, a common and relatively non-toxic solvent which can easily be used in conservation studies.

The hydrodynamic characterisation analyses from sedimentation equilibrium demonstrated that the polymer had a *M*_w_ of ~ 4.3 kDa, reinforced by the approximate value (relative to polymethylmethacrylate standards) obtained by GPC. Sedimentation velocity showed that the polymer system had a degree of heterogeneity, with a high population of a low molar mass species. Conformation analyses indicated that the polymer had an elongated shape, with both *P* and ν giving consistent results and the β-function confirming an elongated prolate rather than a flat oblate disc shape.

The collective properties of our synthesised bioinspired polymer **TPA5** appear to satisfy the essential molecular criteria as a consolidant: high hydrogen bonding potential enhanced by an elongated shape, together with a small injectable molar mass which can be cured into larger, stronger and stable structures once infused or injected into the wood, suggesting this polymer is worthy of further consideration as a potential consolidant.

In future papers in this series we will explore the wood penetration ability of this and related polymers and the ability, after curing, to form a strong stable hydrogen bonded network with porous, degraded wood structures. The interactions with other materials such as calcium hydroxide nanoparticles, used to lower the acid levels in archaeological wood^51^, and their ability to interact in a compatible way with other consolidants, such as the sheet like lignin molecules^70^ will be also be explored. In archaeological wood, lignin has a much slower degradation profile than cellulose, so consolidants should ideally be able to network or interact with the remaining lignin within the wood.

## Materials and methods

### Materials

All reagents and solvents were purchased from a chemical supplier (Acros Organics, Alfa Aesar, Merck, Sigma Aldrich or Fischer Scientific UK) and used without further purification. Water was deionised before use. Brine is a saturated aqueous solution of sodium chloride. Thin layer chromatography was performed on silica gel mounted on aluminium and was visualised using a potassium permanganate dip with gentle heating. Rotary evaporators under reduced pressure were used for solvent evaporation. Dry solvents were obtained from solvent drying towers and contained < 17 parts per million (ppm) of water. Experiments carried out under an inert atmosphere employed argon by means of a Schlenk line or a balloon.

### Nuclear magnetic resonance

^1^H NMR spectra were recorded in deuterated chloroform (CDCl_3_), deuterated DMSO ((CD_3_)_2_SO) and deuterated methanol (CD_3_OD) at ambient temperature using Bruker 400 MHz spectrometers (Bruker Corporation, Germany). ^13^C NMR spectra were recorded in CDCl_3_, (CD_3_)_2_SO and CD_3_OD at ambient temperature using 100 MHz spectrometers. Data is expressed as chemical shifts (δ) in ppm relative to solvent signals (C*H*Cl_3_, ^1^H NMR 7.26), (*C*DCl_3_^13^C NMR 77.16), ((C*H*_3_)_2_SO, ^1^H NMR 2.50), ((*C*D_3_)_2_SO, ^13^C NMR 39.52), (C*H*_3_OH, ^1^H NMR 3.31) or (*C*D_3_OD, ^13^C NMR 49.0) as the internal standard. MestReNova 6.0.2 copyright 2009 (Mestrelab Research S. L.) was used for analysing the spectra.

### High resolution mass spectrometry

Electrospray ionisation (ESI) high-resolution mass spectrometry (HRMS) analyses were performed on a Bruker micrOTOFII mass spectrometer (Bruker Daltonik, Bremen, Germany), interfaced to an Agilent 1200 HPLC (Agilent Technologies, Santa Clara, USA). Samples were presented in solution for analysis by Flow Injection, 1 μL of solution being injected into the ion source of the instrument along with a flow of 0.2 mL min^−1^ of 70% methanol/water eluent. The mass spectrometer was operated in electrospray ionisation (ESI) mode at a typical resolving power of 8000. Control of the analysis was performed through Bruker’s Compass Open Access QC automated data acquisition and reporting software (v1.3; Bruker Daltonik, Bremen, Germany).

### Fourier-transform infra-red spectroscopy.

A Bruker Tensor 27 FT-IR spectrophotometer with an ATR attachment was employed. The measurements were performed in the range of 4000–650 cm^−1^ and spectra were analysed using OPUS software (Bruker Corporation, Germany).

### Monomer and polymer synthesis

The synthesis of α-pinene oxide (**1**), *trans*-sobrerol (**2**) and the triol (**3**) followed the methods described by Thomsett et al.^46^ and are detailed in the Supplementary Information.

#### Synthesis of triol acrylate, 4

**3** (2 g, 10.6 mmol) and butylated hydroxytoluene (BHT) (ca. 19 mg) were added to acetonitrile (13 mL). Triethylamine (4.5 mL, 32 mmol) was added to the reaction mixture. The solution was cooled to 0 °C, after which propylphosphonic anhydride (T3P) (50 wt. % in ethyl acetate, 7.6 mL, 12.8 mmol) and acrylic acid (acid with low H_2_O content, 99.5% stab. with ca. 200 ppm methoxyphenol, 0.8 mL, 11.7 mmol) were added. The mixture was left to stir for 10 min and then allowed to warm to room temperature. The mixture was left stirring for a further 24 h and then concentrated under reduced pressure. The crude product was purified by column chromatography to yield the title compounds as a white solid (**4a** and **4b**) (1.10 g, **4a**:**4b** = 1.3:1, 4.54 mmol, 43% yield). The mixture of diastereomers was partially separated by column chromatography to obtain a pure sample of the major diastereomer **4a** (0.50 g, 2.1 mmol, 19% yield).

#### General procedure for the polymerisation of triol acrylate, 4, to yield a terpene-derived acrylated polymer, TPA5

1,4-dioxane (25 mL) was added to triol acrylate (with a diastereomeric ratio of **4a**:**4b** = 1.3:1) (515 mg, 2.2 mmol) and azobisisobutyronitrile (AIBN) (2.5 mg, 0.015 mmol). The mixture was purged with argon for 45 min, after which it was stirred for 18 h at 70.0 °C under argon. The mixture was left to cool to room temperature and then mixed with 80 mL of acetonitrile to precipitate a white solid. The solution was decanted and the solid dried in a high vacuum oven to yield the title compound (**TPA5**).

### Hydrodynamic characterisation studies

#### Gel permeation chromatography (GPC)

An Agilent 1260 Infinity Series HPLC (Agilent Technologies, USA) fitted with a differential refractive index detector (DRI) was used. THF (HPLC grade, Fisher Scientific) was used as the eluent at room temperature with two Agilent PL-gel mixed-E columns in series at a flow rate of 1 mL min^−1^. A calibration curve was made using polymethylmethacrylate standards with ASTRA software (Wyatt Technology, USA). This was used for the determination of the *M*_n_, *M*_w_ and molar mass distribution (dispersity, *Đ* = *M*_w_/*M*_n_).

### Density measurements—calculation of the partial specific volume $\bar v$

An Anton Paar DMA 5000 V5.003 was used at 20.0 °C. A 9.0 mg/mL stock solution of TPA5 in isopropanol was prepared and then diluted to 8.0, 7.0, 6.0, 5.0 and 4.0 mg/mL. These concentrations were thereafter used for the density measurements. The partial specific volume—needed for the sedimentation velocity and equilibrium experiments—was evaluated following the procedure of Kratky et al.^53^.

### Analytical ultracentrifugation (AUC)

Two Beckman Optima XL-I analytical ultracentrifuges with Rayleigh interference optics were used at 20.0 °C. 12 mm optical path length double sector cells with titanium centrepieces were employed.

#### Sedimentation equilibrium

Loading concentrations of 0.5 to 4 mg/mL of **TPA5** in isopropanol were used. 100 μL of each concentration were injected in the sample solution channel of the AUC cell. Isopropanol was used as the reference solution. The experiment was carried out at a rotational speed of 45,000 rpm over 2 days. The results were analysed with SEDFIT-MSTAR^55^ in order to obtain the *M*_w,app_, making use of the M* function and extrapolation^56^ and the hinge point method. No significant concentration dependence was observed suggesting that non-ideality was not significant. The data obtained from the highest concentration (4.0 mg/ml) was additionally analysed with the MULTISIG algorithm^57^ to evaluate the molar mass distribution.

#### Sedimentation velocity

405 μL of the previous loading concentrations (0.5 to 4.0 mg/mL) of **TPA5** in isopropanol were added to each of the cells. A rotational speed of 49,000 rpm was used and the samples centrifuged overnight. The weight average sedimentation coefficient and the distributions of sedimentation coefficient *c(s)* vs s were obtained by analysis with the SEDFIT procedure^61^.

### Viscosity measurements—calculation of the intrinsic viscosity

An Anton-Paar AMVn (Graz, Austria) rolling ball viscometer was used at a temperature of 8.0 °C. Its closed capillary system is more suitable for working with volatile solvent systems compared to conventional Ostwald viscometers.

The viscosity measurements were carried out using a 6.0 mg/mL concentration of **TPA5** in isopropanol. The intrinsic viscosity was then calculated with the Solomon-Ciuta equation:

6 $$\left[\eta \right]= \frac{1}{c}{\left(2\left({\eta }_{sp}\right)-2ln({\eta }_{r})\right)}^\frac{1}{2}$$

### Conformation analyses

The ELLIPS1^62^ algorithm was used to carry out analyses on the Perrin function (*P*), viscosity increment (ν) and the hydration independent Scheraga-Mandelkern ($\beta$) shape factors.

## Supplementary Information

Supplementary Information
