# Supplementary material for: Terpene polyacrylate TPA5 shows favorable molecular hydrodynamic properties as a potential bioinspired archaeological wood consolidant
Source: Sci Rep. 2021 Apr 1;11:7343. doi: 10.1038/s41598-021-86543-1 (PMC8016987; doi:10.1038/s41598-021-86543-1)
Supplement: Supplementary file 1 — Supplementary Information [file 41598_2021_86543_MOESM1_ESM.docx]

**Terpene polyacrylate TPA5 shows favorable molecular hydrodynamic properties as a potential bioinspired archaeological wood consolidant**

Michelle Cutajar^1,2*^, Fabrizio Andriulo^3^, Megan R. Thomsett^2^, Jonathan C. Moore^2^, Benoit Couturaud^4^, Steven M. Howdle^2^, Robert A. Stockman^2*^ and Stephen E. Harding^3,1*^

1. *National Centre for Macromolecular Hydrodynamics (NCMH), University of Nottingham, School of Biosciences, Sutton Bonington, LE12 5RD, U.K.;*
2. *School of Chemistry, University of Nottingham, University Park Nottingham, NG7 2RD;*
3. *Museum of Cultural History, University of Oslo, Postboks 6762, St. Olavs plass, 0130 Oslo, Norway*
4. *Univ Paris Est Creteil, CNRS, Institut de Chimie et des Matériaux Paris-Est (ICMPE), UMR 7182, 2 rue Henri Dunant, 94320 Thiais, France*

**Supplementary Material**

**α-Pinene oxide (1)**

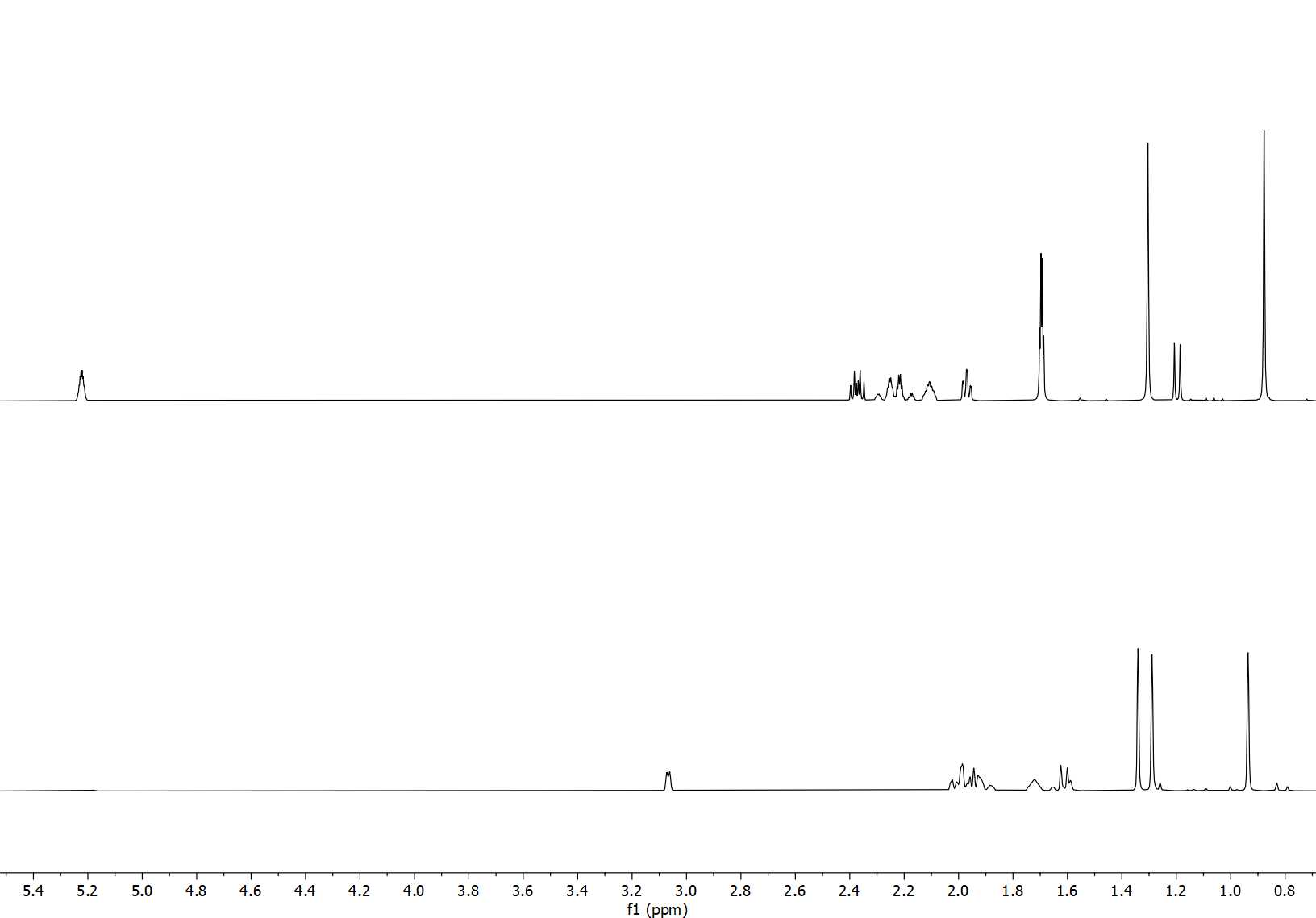


*Synthesis of α-pinene oxide,* ***1****.* 1S-(-)-α-pinene (5.82 mL, 36.7 mmol) was added to a suspension of NaHCO_3_ (3.92 g, 46.6 mmol) in CH_2_Cl_2_ (7.5 mL) and then cooled to 0 °C. *Meta*-chloroperbenzoic acid (*m*CPBA) (~70%, 9.22 g, 37.4 mmol) was gradually added to the solution. The reaction was stirred for 1 hour, after which saturated aqueous solution of Na_2_SO_3_ (27 mL) was added to the reaction mixture. The reaction was allowed to settle to room temperature and stirred for a further 30 minutes. The reaction mixture was diluted with saturated aqueous solution of NaHCO_3_ (30 mL) and CH_2_Cl_2_ (60 mL). The aqueous washings were extracted with CH_2_Cl_2_ (75 mL). The organic phase was washed with saturated aqueous solution of NaHCO_3_ (3 x 100 mL). The organic extracts were then combined, washed with brine (3 x 100 mL), dried over MgSO_4_, filtered and concentrated under reduced pressure to yield the title compound (**1**) (4.78 g, 31.4 mmol, 86% yield).

**FTIR** (ATR) ν_max_ /cm^-1^: 2977, 2914, 2834, 1229, 1084, 943, 818; **^1^H NMR** (400 MHz, CDCl_3_) δ_H_ 3.07 (d, *J* = 4.1 Hz, 1H), 2.01 – 1.83 (m, 4H), 1.72 (s, 1H), 1.61 (d, *J* = 9.4, 1H), 1.34 (s, 3H), 1.29 (s, 3H), 0.94 (s, 3H); **^13^C NMR** (100 MHz, CDCl_3_) δ_C_ 60.3, 56.9, 45.1, 40.5, 39.7, 27.6, 26.7, 25.9, 22.4, 20.2.

***Trans*-sobrerol (2)**

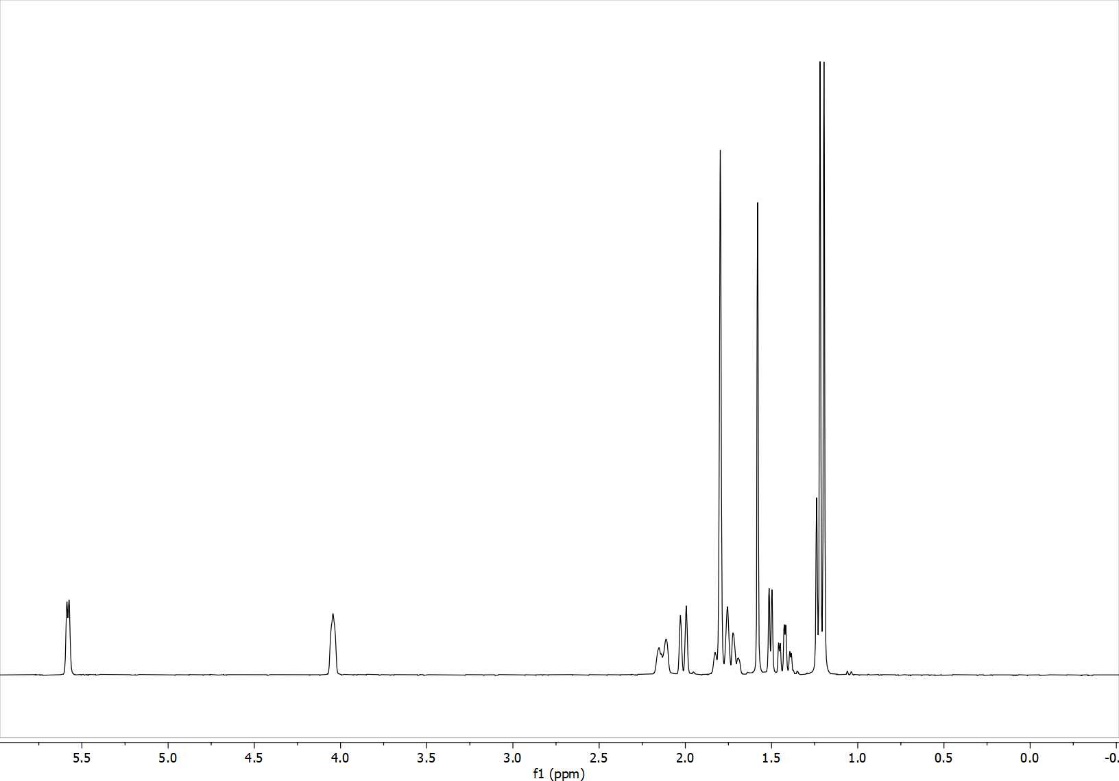


*Synthesis of trans-sobrerol,* ***2****.* CO_2_ was continuously passed through H_2_O (52 mL) until the pH was approximately 4.5 – 5. **1** (4 g, 26.3 mmol) was then added and the mixture stirred at room temperature for 24 hours. The solution was concentrated under reduced pressure and a white solid precipitated. The crude solid was washed with cold ethyl acetate (2 x 5 mL) to give the title compound as a white, crystalline solid (**2**) (2.44 g, 14.4 mmol, 55% yield).

**FTIR** (ATR) ν_max_ /cm^-1^: 3321, 2973, 2887, 1376, 1052, 919; **^1^H NMR** (400 MHz, CDCl_3_) δ_H_ 5.58 (d, *J* = 5.4 Hz, 1H), 4.04 (s, 1H), 2.17 – 2.08 (m, 1H), 2.05 – 1.97 (m, 1H), 1.84 – 1.67 (m, 5H), 1.42 (td, *J* = 13.1, 3.9 Hz, 1H), 1.22 (s, 3H), 1.19 (s, 3H); **^13^C NMR** (100 MHz, CDCl_3_) δ_C_ 133.2, 126.6, 71.3, 68.8, 38.9, 33.8, 27.8, 27.3, 26.5, 21.0; **HRMS** (ESI) m/z calculated for [C_10_H_18_NaO_2_]^+^ 193.1204 found 193.1210 (M^+^ Na^+^).

**(1*S*,2*S*)-5*t*-(2-hydroxypropan-2-yl)-2*c*-methylcyclohexane-1*r*,3*t*-diol (3)**

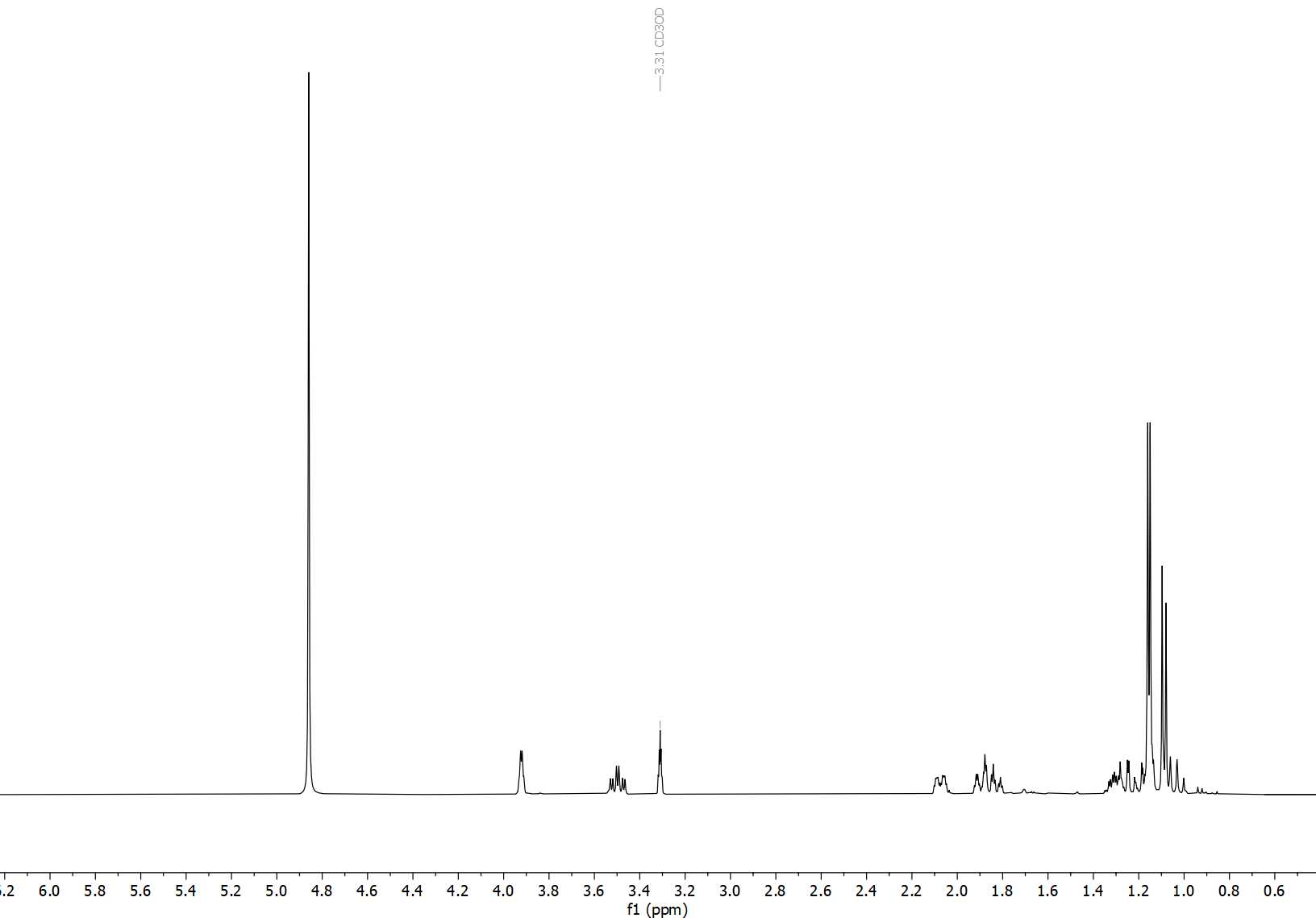


*Synthesis of triol,* ***3***. A solution of **2** (2.17 g, 12.8 mmol) in dry THF (13 mL) was cooled to 0 °C and BH_3_.THF (1M in THF) (26 mL, 25.5 mmol) was added dropwise over 30 minutes. The reaction mixture was stirred for 90 minutes, after which NaOH (2 M, 19 mL) and H_2_O_2_ (30%, 3.9 mL) were added dropwise. The mixture was allowed to warm to room temperature and left to stir for 16 hours. Afterwards, saturated aqueous solution of Na_2_SO_3_ (50 mL) was added and stirred for 15 minutes. The mixture was concentrated under reduced pressure and the resulting solid was dissolved in hot acetonitrile (85 °C). The solid was removed by filtration while the solution was hot. The filtrate was concentrated under reduced pressure and recrystallised from acetonitrile to yield the title compound (**3**) (1.32 g, 7.01 mmol, 55% yield).

**FTIR** (ATR) ν_max_ /cm^-1^: 3298, 2975, 2886, 1276, 1082, 911; **^1^H NMR** (400 MHz, CD_3_OD) δ_H_ 3.92 (q, *J* = 3.0 Hz, 1H), 3.50 (td, *J* = 4.3 Hz, 1H), 2.11 – 2.04 (m, 1H), 1.94 – 1.86 (m, 1H), 1.87 – 1.79 (m, 1H), 1.35 – 1.26 (m, 1H), 1.25 (d, *J* = 2.6 Hz, 1H), 1.16 (s, 3H), 1.15 (s, 3H), 1.09 (m, 3H), 1.07 (m, 1H); **^13^C NMR** (100 MHz, CD_3_OD) δ_C_ 72.7, 72.7, 72.2, 45.1, 42.5, 37.7, 35.6, 27.4, 26.8, 14.9; **HRMS** (ESI) m/z calculated for [C_10_H_18_NaO_2_]^+^ 193.1204, found 193.1198 (M + Na^+^).


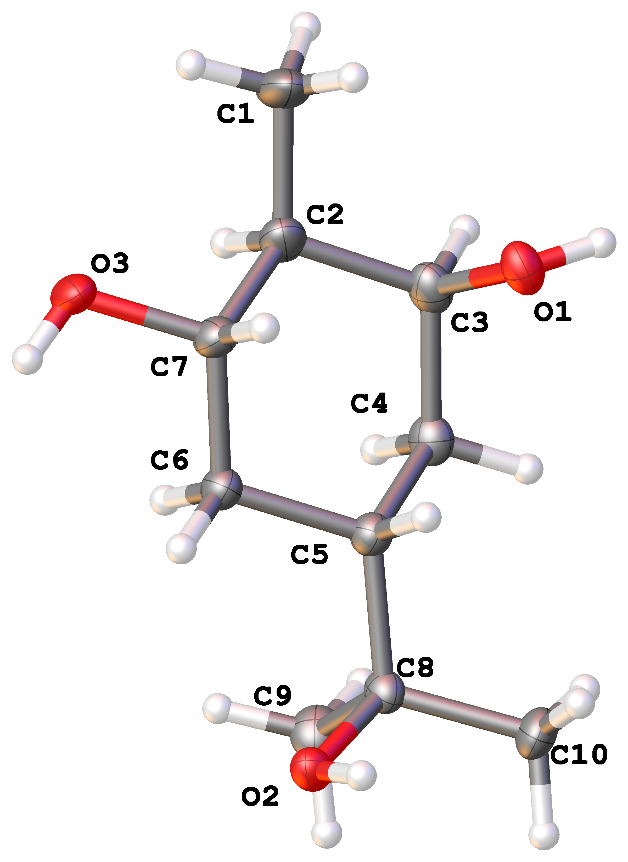


X-Ray structure of the major diastereomer. CCDC#1893327

| **Crystal data and structure refinement.** | |
| --- | --- |
| Identification code | MRTRSB |
| Empirical formula | C_10_H_20_O_3_ |
| Formula weight | 188.26 |
| Temperature/K | 120(2) |
| Crystal system | triclinic |
| Space group | P-1 |
| a/Å | 10.7645(6) |
| b/Å | 12.5124(5) |
| c/Å | 16.7246(7) |
| α/° | 78.861(3) |
| β/° | 79.889(4) |
| γ/° | 72.560(4) |
| Volume/Å^3^ | 2091.88(18) |
| Z | 8 |
| ρ_calc_g/cm^3^ | 1.196 |
| μ/mm^‑1^ | 0.698 |
| F(000) | 832.0 |
| Crystal size/mm^3^ | 0.2531 × 0.1723 × 0.0385 |
| Radiation | CuKα (λ = 1.54184) |
| 2Θ range for data collection/° | 7.49 to 147.984 |
| Index ranges | -13 ≤ h ≤ 13, -15 ≤ k ≤ 15, -20 ≤ l ≤ 20 |
| Reflections collected | 8269 |
| Independent reflections | 8269 [R_int_ = ?, R_sigma_ = 0.0298] |
| Data/restraints/parameters | 8269/0/495 |
| Goodness-of-fit on F^2^ | 1.551 |
| Final R indexes [I>=2σ (I)] | R_1_ = 0.1025, wR_2_ = 0.3378 |
| Final R indexes [all data] | R_1_ = 0.1074, wR_2_ = 0.3455 |
| Largest diff. peak/hole / e Å^-3^ | 1.34/-0.79 |

**(1*R*,2*R*,3*R*,5*R*)-3-Hydroxy-5-(2-hydroxypropan-2-yl)-2-methylcyclohexyl acrylate (4a) and (1*R*,2*S*,3*R*,5*S*)-3-Hydroxy-5-(2-hydroxypropan-2-yl)-2-methylcyclohexyl acrylate (4b)**

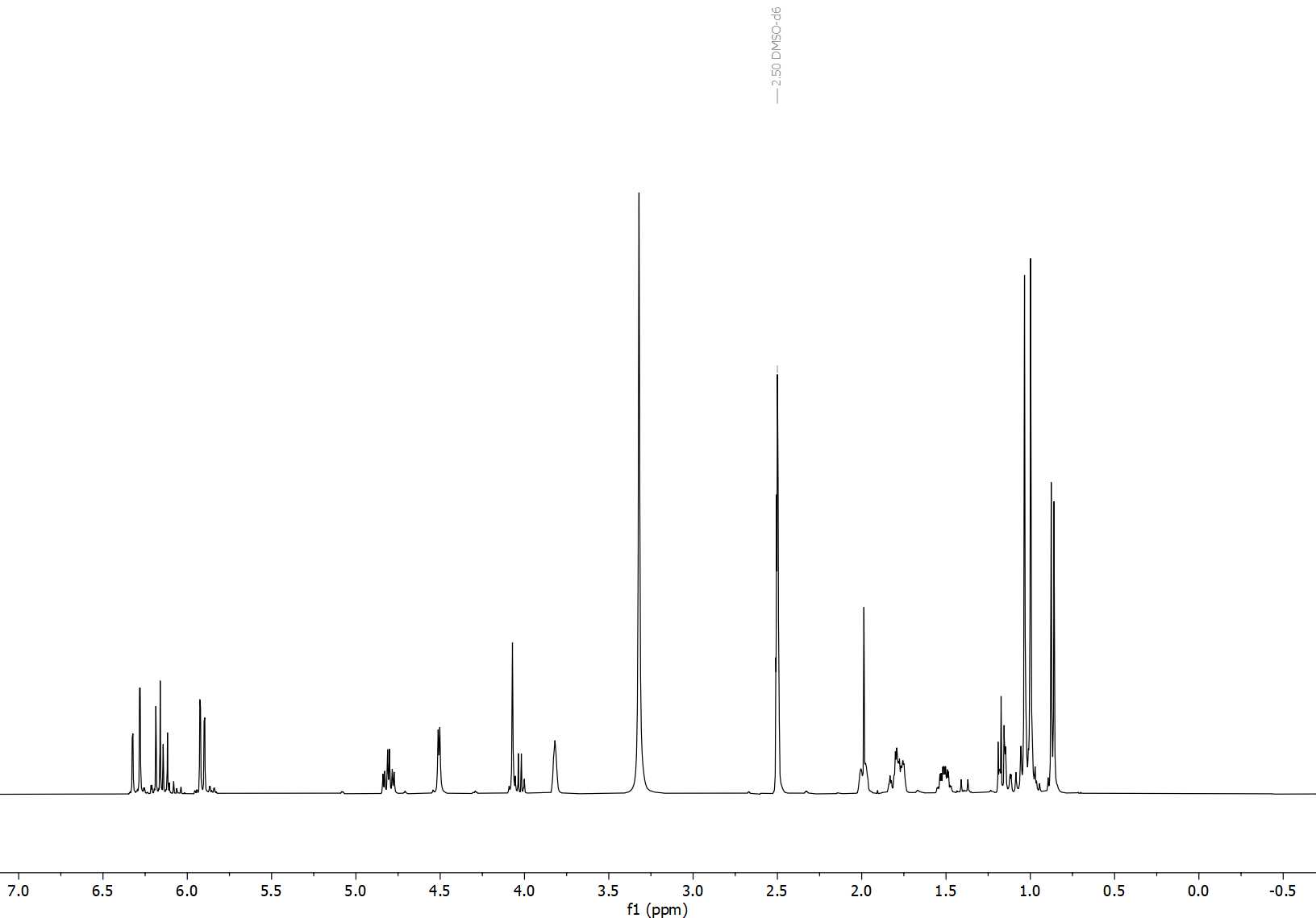


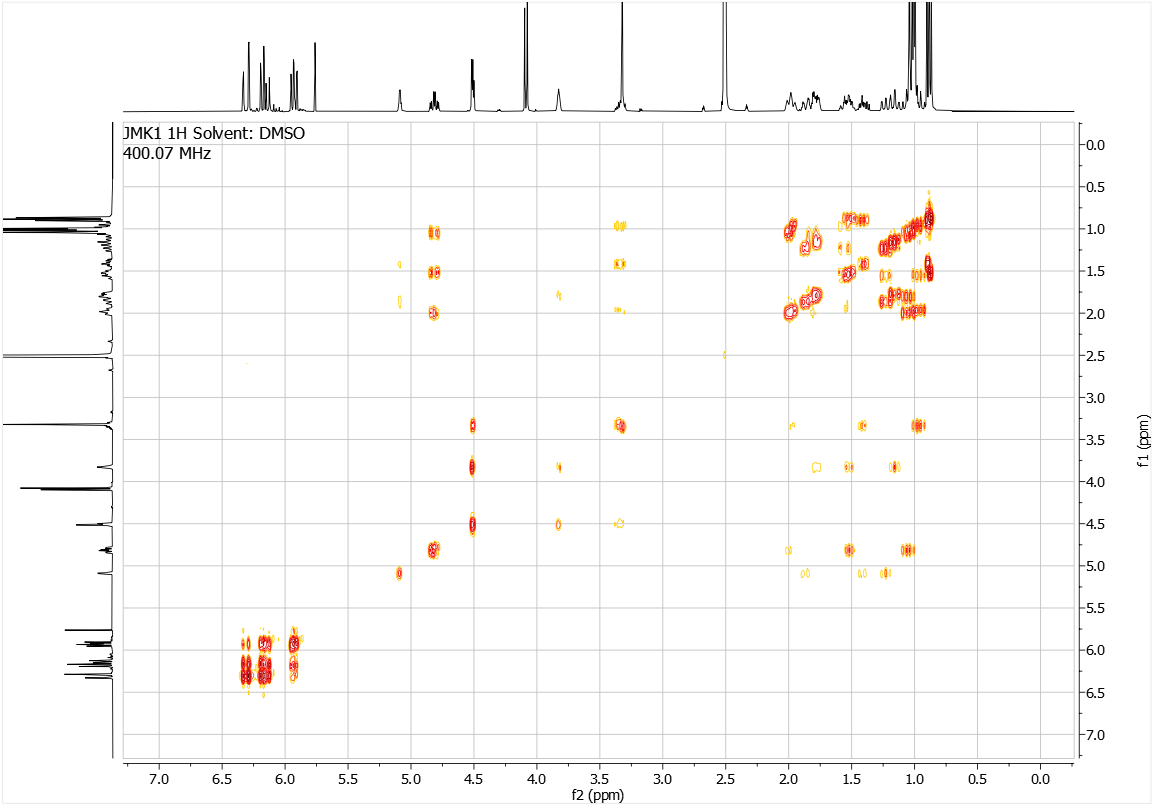


Only the peaks for the major diastereomer (**4a**) are reported.

**FTIR** (ATR) ν_max_ /cm^-1^: 3340, 2975, 2934, 2874, 1720, 1625, 1404, 1295, 1271, 1190; **^1^H NMR** (400 MHz, (CD_3_)_2_SO) δ_H_ 6.30 (dd, *J* = 17.3, 1.7 Hz, 1H), 6.15 (dd, *J* = 17.3, 10.2 Hz, 1H), 5.91 (dd, *J* = 10.3 Hz, 1H), 4.81 (td, *J* = 11.1, 4.4 Hz, 1H), 4.51 (d, *J* = 4.1 Hz, 1H), 4.07 (s, 1H), 3.82 (m, 1H), 1.99 (dt, *J* = 11.8, 4.8 Hz, 1H), 1.79 (m, 2H), 1.51 (dtt, *J* = 13.3, 6.5, 3.6 Hz, 1H), 1.02 (d, *J* = 14.4 Hz, 6H), 0.87 (d, *J* = 6.7 Hz, 3H); **^13^C NMR** (100 MHz, (CD_3_)_2_SO) δ_C_ 165.0, 131.1, 127.8, 75.6, 69.9, 66.3, 42.8, 41.7, 36.7, 29.8, 27.2, 26.2,14.9; **HRMS** (ESI) m/z calculated for C_13_H_22_NaO_4_ [M+Na]^+^ 265.1416 found 265.1427.

**Polymer TPA5**

**FTIR** (ATR) ν_max_ /cm^-1^: 3370, 2967, 2928, 2880, 2114, 1717, 1454, 1368, 1368, 1256, 1168, 1122, 1098, 916, 683; **^1^H NMR** (400 MHz, (CD_3_)_2_SO) δ_H_ 4.98 (m, 1H), 4.70 (m, 1H), 4.44 (m, 1H), 4.05 (m, 1H), 3.79 (m, 1H), 2.23 (m, 1H), 1.94 (m, 1H), 1.78 (m, 2H), 1.41 (d, *J* = 50.6 Hz, 3H), 1.02 (m, 10H), 0.87 (m, 4H).

**Supplementary Figure S1** Reaction monitoring of the polymerisation reaction using ^1^H NMR and GPC in THF


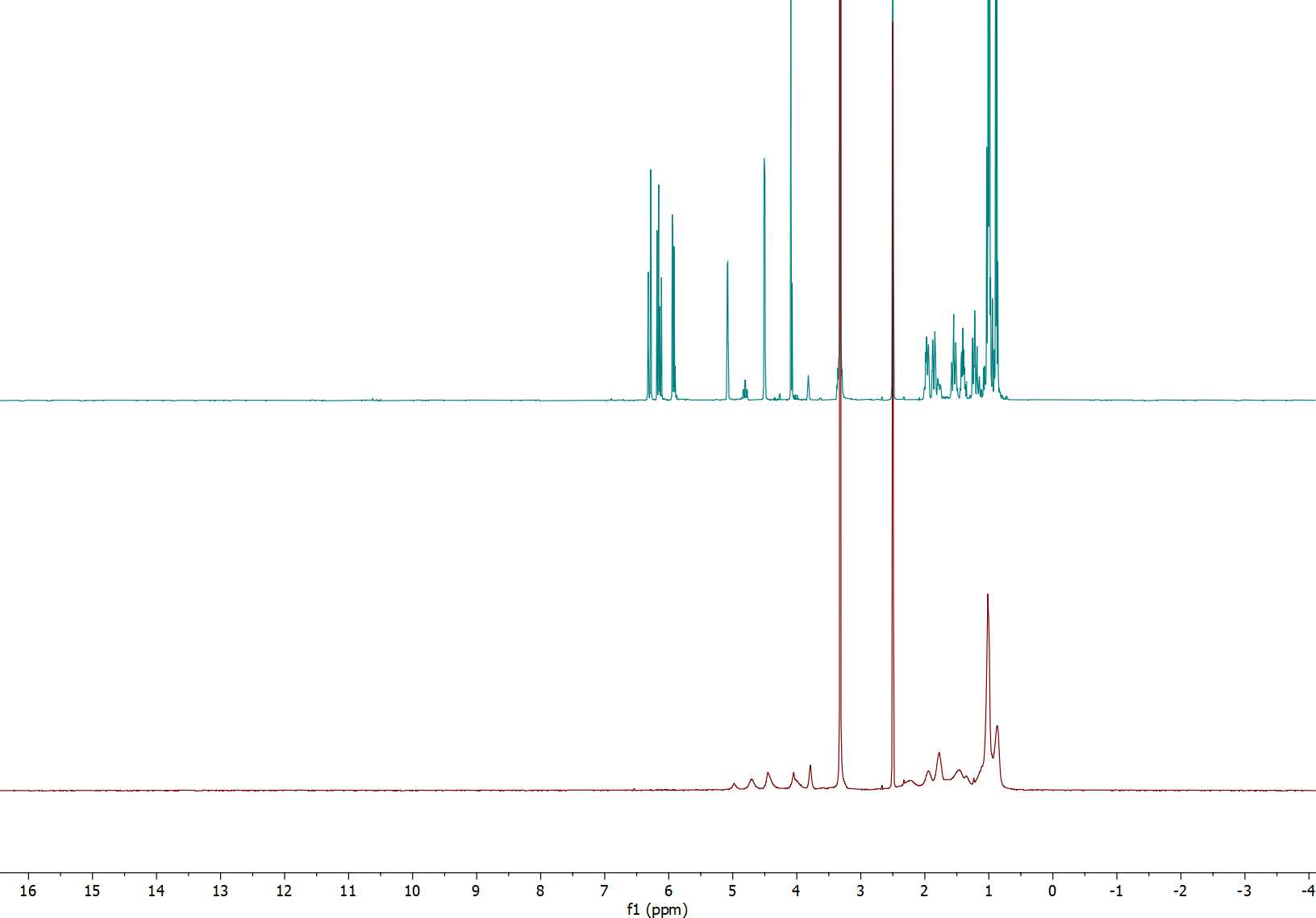

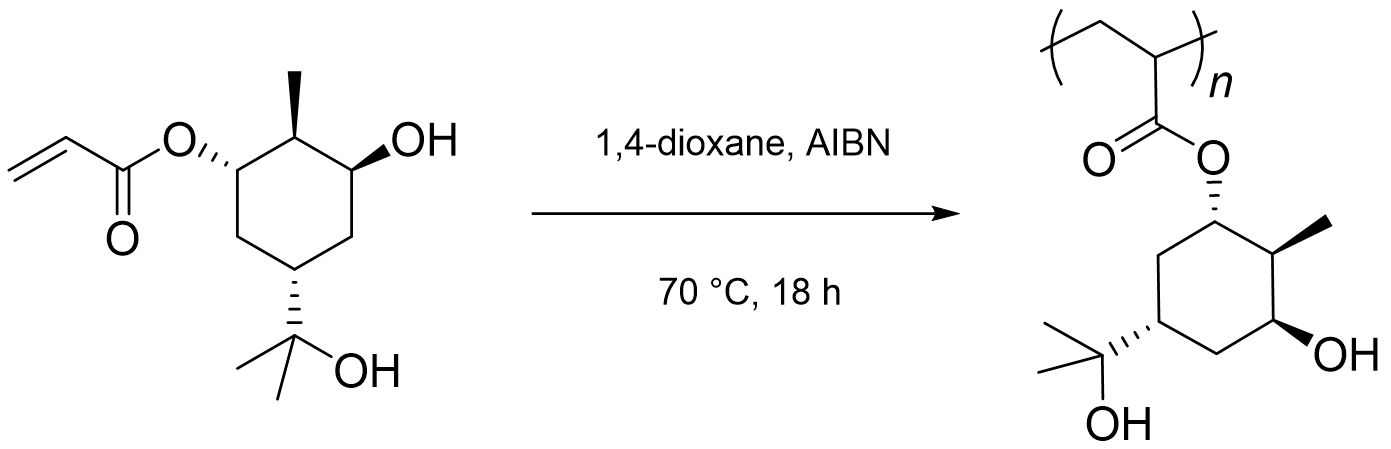

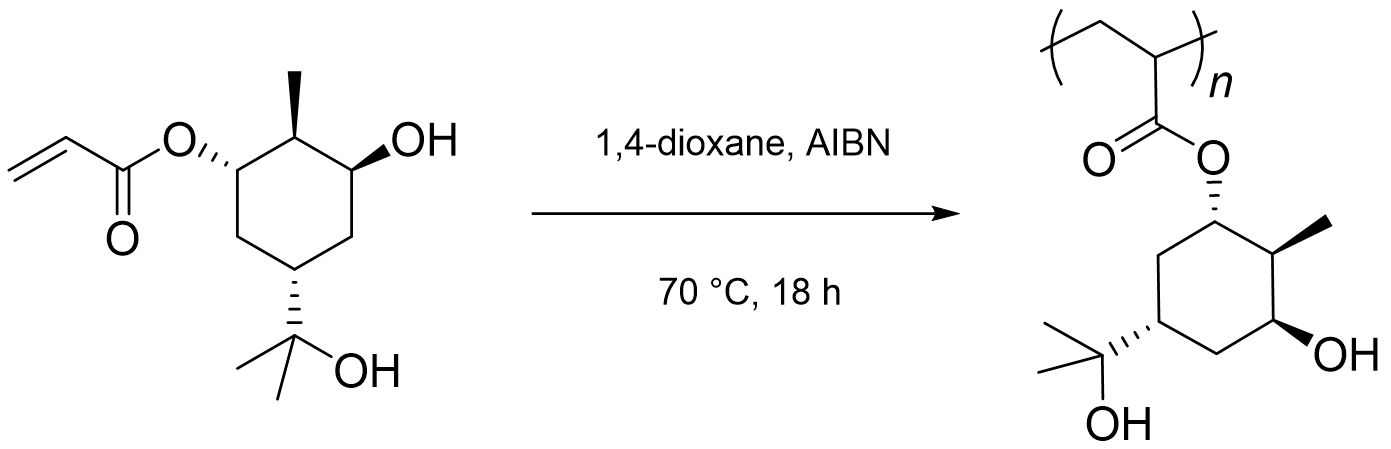


**Supplementary Figure S2** Comparison of the ^1^H NMR spectra of the monomer with that of the polymer **TPA5**, indicating that the monomer had been consumed
